# Revitalizing child health: lessons from the past

**DOI:** 10.1080/16549716.2021.1947565

**Published:** 2021-07-29

**Authors:** Kathleen L. Strong, Jennifer Requejo, Ambrose Agweyu, Sk Masum Billah, Cynthia Boschi-Pinto, Sayaka Horiuchi, Zeina Jamaluddine, Marzia Lazzerini, Abdoulaye Maiga, Neil McKerrow, Melinda Munos, Joanna Schellenberg, Ralf Weigel

**Affiliations:** a Maternal, Newborn, Child and Adolescent Health and Aging Department, World Health Organization, Geneva, Switzerland; b Division of Data, Analytics, Planning and Monitoring, UNICEF, New York, NY, USA; c Department of Epidemiology and Demography, KEMRI-Wellcome Trust Research Programme, Kenya; d Maternal and Child Health Division, Icddr, b, Dhaka, Bangladesh; e Department of Epidemiology and Biostatistics, University Federal Fluminense Rio De Janeiro, Brazil; f Center for Birth Cohort Studies, University of Yamanashi, Yamanashi, Japan; g Epidemiology and International Health, LSHTM, London, UK; h Center for Maternal and Child Health, Institute for Maternal and Child Health IRCCS Burlo Garofolo, Trieste, Italy; i Global Disease epidemiology and control, Johns Hopkins Bloomberg School of Public Health, Baltimore, MD, USA; j Department of Paediatrics and Child Health, Nelson R Mandela School of Medicine, University of KwaZulu-Natal, Durban, South Africa; k Department of Paediatrics and Child Health, University of Cape Town, Rondebosch, South Africa; l Global Child Health, Witten/Herdecke University, Witten-Herdecke, Germany

**Keywords:** child health and well being; child mortality; global public health initiatives; epidemiology

## Abstract

Essential health, education and other service disruptions arising from the COVID-19 pandemic risk reversing some of the hard-won gains in improving child survival over the past 40 years. Although children have milder symptoms of COVID-19 disease than adults, pandemic control measures in many countries have disrupted health, education and other services for children, often leaving them without access to birth and postnatal care, vaccinations and early childhood preventive and treatment services. These disruptions mean that the SARS-CoV-2 virus, along with climate change and shifting epidemiological and demographic patterns, are challenging the survival gains that we have seen over the past 40 years. We revisit the initiatives and actions of the past that catalyzed survival improvements in an effort to learn how to maintain these gains even in the face of today’s global challenges.

## Background

More children are surviving past the age of 5 years than ever before because essential services, including immunization against childhood diseases, nutrition support and sick child visits, were prioritized by global health initiatives seeking to eliminate preventable deaths in children under 5 years. In many countries these services were enhanced and supported by improvements in other policy areas, including education, environment, health systems, health financing and a health workforce [1]. During the Covid-19 pandemic, these essential health services and the systems, financing and workforce that support them have been disrupted globally because of facility closures, transportation limitations, family fear of attending clinics and reorganization of health services to focus on COVID-19 patient care. World Health Organization (WHO) pulse surveys of key informants in countries in 2020 and again in early 2021 show that 93% of 187 countries suffered at least some service disruptions at the start of the pandemic, falling slightly to 89% of 135 countries by early 2021 [2]. High-income countries were less affected than low- and middle-income countries. The most frequently reported service disruptions were for contraception and family planning and management of moderate-to-severe malnutrition. Immunization services, both routine facility based and outreach, also suffered disruptions, and one-third of all reporting countries still have disruptions to these services as of early 2021 [2].

To rebuild and strengthen child health services in the wake of the COVID-19 pandemic, we must first understand the evolution of the child health initiatives that catalyzed past action to improve child survival, assess the relevance of those efforts for the current decade, prioritize actions and interventions, and reinforce the mechanisms used to monitor the impact. This paper reviews the sequence of public health actions specific to the child health landscape over the past 40 years to learn lessons that will inform how to move forward in support of the UN Sustainable Development Goals (SDGs) of 2030 and leave no child behind.

## Child health initiatives evolved from a vertical program focus to integrated management of childhood disease

**Figure 1.**
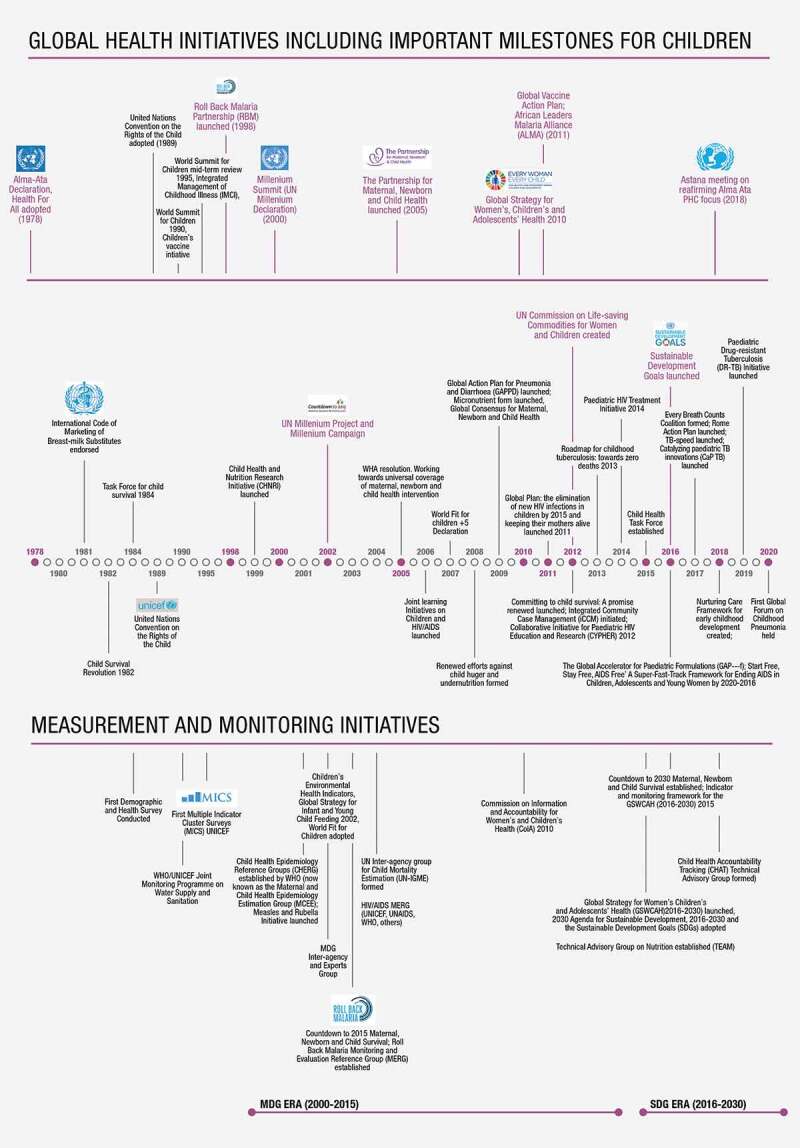
Summary and timeline for global public health initiatives focused on children

The Alma Ata declaration of *Health for All* in 1978 emphasized the right to health for all populations regardless of geographic location. For children, this declaration was followed up in 1979 with the United Nations Educational, Scientific and Cultural Organization’s (UNESCO) announcement of the International Year of the Child to draw attention to issues that affected children all over the world. This ushered in four decades of action for child health and well-being as described in Figure 1.

In the 1980s, child survival was tied closely to adequate and appropriate infant and young child feeding, disease prevention through vaccines, treatment of common childhood illnesses such as diarrhoea, pneumonia and malaria, and improvements in access to clean water and improved sanitation facilities. Global initiatives in this decade geared up to improve child survival through vertical programs tied to single issues, such as nutrition, immunization and treating infectious diseases of childhood. Examples of actions during this period include the endorsement of the International Code on Marketing of Breastmilk Substitutes in 1981, the Child Survival Revolution in 1982 and the Task Force for Child Survival in 1984 (Figure 1). These actions focused on child under-5 survival at a time when the global under-5 mortality rate was approximately 60% higher than it is today. The decade ended with the adoption of the United Nations Convention on the Rights of the Child in 1989, strongly asserting that health and well-being are rights for all children, everywhere.

Initiatives rolled out in the 1990s included the UN World Summit for Children and the Children’s Vaccine Initiative, both launched in 1990. The World Summit for Children was intended as a (Plan of Action) for national governments, international organizations, non-governmental organizations (NGOs) and donors following the UN Convention on the Rights of the Child. The action plan focused on child survival, protection and development. It included a goal for child survival to be achieved by all countries and formulated as ‘a reduction of 1990 under-5 child mortality rates by one third or to the level of 70 per 1,000 live births by 2000, whichever is the greater reduction’ [3].

Movement towards an integrated management of childhood illness gained momentum so that by the mid-1990s integrated management of childhood illnesses, including by improved nutrition and immunization, was the preferred strategy for combating infectious diseases of childhood. For example, with *Alma Ata*, WHO shifted away from a vertical cholera-focused program to a program that addressed all diarrhoeal diseases (the Program for the Control of Diarrhoeal Diseases (CDD)), which in the 1990s became more comprehensive, combining research with country implementation activities [4]. The success of CDD reinforced the idea that treating the leading childhood illnesses, pneumonia, diarrhoea and malaria, together using clear guidance for managing childhood illnesses in communities was the way to improve child survival. To this end, WHO, UNICEF and collaborators created the Integrated Management of Childhood Illness (IMCI), which includes three components covering case management skills of health-care workers, the readiness of the health-care system, and family and community health practices [5]. IMCI presents guidelines for the combined treatment of major childhood illnesses, emphasizing prevention of disease through immunization and improved nutrition. Later iterations included a name change to IMNCI to add specific guidance for newborns and more details on implementation of the community component.

The Millennium Development Goals (MDGs) were set for the period from 2000 to 2015 and focused on reduction of maternal and child mortality. The SDGs set in 2015 included recognition of the importance of the broader context on child well-being and development and a focus on quality of care and equitable health outcomes. Measurement and monitoring of results for the MDGs and SDGs have strengthened indicator frameworks and efforts to standardize data collection and reporting mechanisms on mortality, disease incidence and prevalence, health intervention coverage and quality of care. The 2005 World Health Assembly resolution *Working towards universal coverage of maternal, newborn and child health interventions* changed the language of child health initiatives, moving towards the inclusion of women and adolescents alongside children in recognition that a broader, integrated perspective helps to continue the gains in child survival throughout the course of life. This combination is seen in the launch of the first Every Woman, Every Child Global Strategy for Woman’s, Children’s and Adolescents’ Health (EWEC GS) in 2010, and repeated in the launch of the second EWEC GS in 2015 [6]. Recognition of the interlinkages between maternal, newborn, child and adolescent health, however, fostered age-specific initiatives such as the Every Newborn Action Plan [7], Nurturing Care Framework [8], and the Global Accelerated Action for the Health of Adolescents (AA-HA!) [9]. Although these initiatives drew needed attention to newborn health, early childhood development and adolescent health, they also arguably contributed to fragmentation on the global landscape and decreased visibility of child health. Child health continues to be supported mainly through vertical initiatives such as immunization, high burden diseases such as HIV, TB and malaria, and common childhood illnesses such as pneumonia and diarrhoea. Donors and government funding actions may hinder uptake of comprehensive, integrated child health strategies, especially programs that can integrate well and sick child services.

## Measurement and monitoring initiatives supported advocacy and focused action where it was needed most

In 1989, the first attempts to systematically measure health outcomes for women and children took place with the USAID-funded Demographic and Health Surveys (DHS). These population-based surveys ask about births and deaths and have evolved over time to gather more information about health and well-being, ranging from household and family structure to care seeking for common diseases. Shortly afterwards, WHO and UNICEF started the Joint Monitoring Programme on water supply and sanitation, making use of country-led DHS to monitor water and sanitation for health. The launch of the first Multi-indicator Cluster Surveys (MICS) from UNICEF in 1995 was country owned and gave countries the opportunity to lead survey development by adding additional modules of indicators to use in their surveys according to national interest.

The efforts of UN interagency groups such as the UN InterAgency Group for Child Mortality Estimation (UN-IGME)^1^^1^A collaboration of WHO, UNICEF, The World Bank group and the UN Division of Economic and Social Affair’s Population Division, which produces annual country-level estimates of newborn, infant, under-5 and, more recently, children, adolescents and young adult (5–24 years) mortality. along with the WHO-established Child Health Epidemiology Reference Group (CHERG)^2^^2^Now called the Maternal and Child Epidemiology Estimation group (MCEE). make it possible to monitor progress by providing the means to measure where improvements have been made and where they are still needed on child survival. Tracking of trends on child mortality and cause of death shows that deaths are highest in the newborn period and that an increasing number of children and adolescents are surviving but bear the burden of injuries, developmental disabilities, non-communicable diseases and poor mental health. Nonetheless, global improvement in child survival has not been equitably distributed across all regions. Sub-Saharan Africa carries over half (53%) of the under-5 mortality burden followed by Central and Southern Asia (23%), while the remaining regions combined account for only 19% of the global burden [10]. Preterm birth and infectious diseases, particularly pneumonia, diarrhoea and malaria, continue to be the leading causes of under-5 mortality and morbidity, particularly in sub-Saharan Africa, the very region that will have the largest population in the world under 20 years by 2050 [11,12].

The shifting epidemiological and demographic patterns that we see in recent data show that while survival of children under 5 has progressed well, other areas have received less attention in the global public health landscape. These emerging issues in child health include the health and well-being of older children and adolescents (5⎯19 years), and children living with physical and developmental disabilities.

## Emerging issues in child health require a multisectoral, ‘whole-of-government’ approach to extend past the under-5 survival agenda and into the child development and well-being era

Evolution of child health initiatives over the past 40 years led to the realization that health and well-being are interconnected at every stage of life and also across generations. Continuum of care packages became a way of integrating service delivery throughout the life-course to reduce maternal, newborn and child deaths [13]. These packages include interventions to be delivered pre-pregnancy, during pregnancy, at childbirth, postnatally, for children and also for water safety and sanitation. Monitoring of some MDG and now SDG indicators tracks the continuum of care and they are also interconnected across partnerships within the health sector and with other government sectors [14]. For example, initiatives such as Ending Preventable Maternal Mortality (EPMM) and Every Newborn Action Plan (ENAP) are health partnerships initiated to raise the profile of maternal, newborn health and stillbirths [15], reflecting a shift in the focus from under-5 mortality prevention towards the maternal and newborn period. This change filled gaps in guidance for the care of small and sick newborns and also addressed the shifting patterns of under-5 mortality, of which 47% now occur in the first 28 days of life [10]. These partnerships express a desire for integrated action but also the concern that there is under-investment in high-risk periods in the human life-course [15]. Nonetheless, multisectoral partnerships are needed in child health to reduce age- and disease-specific funding fragmentation that may hinder the development of a true life-course approach to health planning and programming.

The cumulative effects of risk and protective factors across different time periods in a child’s life are now better understood and call for strategic shifts in policies and health-care services so that they respond to the diverse and changing needs of children and adolescents, wherever they may live [16]. These shifts included expanding the focus from survival of children under 5 to health, nutrition, psychosocial and supportive environments in the first two decades of life, refocusing action on child survival to target age- and region-specific high mortality burdens emphasizing quality care, high coverage of timely interventions and building children’s resilience through responsive care giving, early learning and promoting optimal health, growth and development. These shifts remain relevant today but the COVID-19 pandemic has caused service delivery gaps that remind us that the prevention and treatment services so instrumental in improving child survival need to be maintained with high quality of care everywhere and particularly in low- and middle-income countries (LMICs).

Moving forward in child health programming and its monitoring requires that we address the following: (1) access, quality and coverage of health and other services with equity, (2) national policies, services and data collections that support health and well-being at each stage of the life-course, and (3) a multisectoral, ‘whole-of-government’ and ‘whole-of-society’ approach that engages sectors not directly related to health but responsible for well-being and security. For example, health, nutrition, early learning and education, responsive care and relationships, personal autonomy and resilience, and a safe and secure environment all contribute to the development of children and adolescents into healthy adults, which in turn leads to a healthier next generation. The principle of a life-course approach naturally leads to a broader health agenda, one that includes not only child survival but also actions that allow children to thrive and prepare to contribute to transforming society for future generations. The so-called ‘thrive’ and ‘transform’ agendas also need an integrated, multisectoral approach that allows other government sectors such as education, environment, transportation and law enforcement, among others, to contribute positively to child health programming.

## Actions needed now and into the future

Even though the longer term implications of the SARS-CoV-2 pandemic on child health and development outcomes remain unknown, a continued focus on child under-5 survival is needed to maintain hard-won gains and to achieve the SDGs. Advancing on this agenda means putting into place programmatic approaches that allow flexibility and responsiveness to the specific needs of children and adolescents living in different regional and socio-economic contexts. At the same time, programmatic flexibility needs to be informed by evidence collected, analyzed and disseminated by countries, UN interagency groups and other partners. Balancing the needs of the survival agenda in some country settings with the needs for an expanded child development and well-being agenda in other settings means that a full range of validated, standard indicators is needed to monitor child health into the future.

Lessons from past initiatives indicate that rigorous monitoring is needed to ensure that multisectoral frameworks are implemented as intended and also to help countries identify their own priority areas for policy and programmatic action. In LMICs, this will be possible only with continued financial and technical support for health information management systems that are affordable and efficient so that programmatic outcomes are regularly measured. The donor community should also provide support to country efforts to implement comprehensive, integrated approaches to child health and well-being that are resilient to shocks such as COVID-19. This includes building country capacity to design comprehensive child and adolescent health programs based on epidemiologic and demographic profiles [17] and moving away from vertical and age-specific funding strategies so that children from newborns to adolescents can survive, thrive and reach their potential everywhere.

## Paper context

The COVID-19 pandemic threatens the sustained improvements in child survival because of disruptions to health services that delay timely prevention and treatment of childhood diseases. The evolution of the child health initiatives that catalyzed past action to improve child survival is used as a lesson on how to rebuild after the pandemic. We believe that continued focus on child survival is needed so that survival gains can be sustained and extended after the pandemic.
